# Hypoxia Supports Epicardial Cell Differentiation in Vascular Smooth Muscle Cells through the Activation of the TGFβ Pathway

**DOI:** 10.3390/jcdd5020019

**Published:** 2018-04-13

**Authors:** Jiayi Tao, Joey V. Barnett, Michiko Watanabe, Diana Ramírez-Bergeron

**Affiliations:** 1 Case Cardiovascular Research Institute, Case Western Reserve University School of Medicine, Cleveland, OH 44106, USA; jtao2@rockets.utoledo.edu; 2 Department of Pharmacology, Vanderbilt University Medical Center, Nashville, TN 37232, USA; joey.barnett@vanderbilt.edu; 3 Department of Pediatrics, Rainbow Babies and Children’s Hospital, The Congenital Heart Collaborative, Cleveland, OH 44106, USA; mxw13@case.edu; 4 University Hospitals Harrington-McLaughlin Heart & Vascular Institute, Cleveland, OH 44106, USA

**Keywords:** hypoxia inducible factor, hypoxia, transforming growth factor beta, TGFβ, RhoA/ROCK, epicardial cell, epithelial-to-mesenchymal-transition, vascular smooth muscle cells, coronary vasculature

## Abstract

Epicardium-derived cells (EPDCs) are an important pool of multipotent cardiovascular progenitor cells. Through epithelial-to-mesenchymal-transition (EMT), EPDCs invade the subepicardium and myocardium and further differentiate into several cell types required for coronary vessel formation. We previously showed that epicardial hypoxia inducible factor (HIF) signaling mediates the invasion of vascular precursor cells critical for patterning the coronary vasculature. Here, we examine the regulatory role of hypoxia (1% oxygen) on EPDC differentiation into vascular smooth muscle cells (VSMCs). Results: Hypoxia stimulates EMT and enhances expression of several VSMC markers in mouse epicardial cell cultures. This stimulation is specifically blocked by inhibiting transforming growth factor-beta (TGFβ) receptor I. Further analyses indicated that hypoxia increases the expression level of TGFβ-1 ligand and phosphorylation of TGFβ receptor II, suggesting an indispensable role of the TGFβ pathway in hypoxia-stimulated VSMC differentiation. We further demonstrate that the non-canonical RhoA/Rho kinase (ROCK) pathway acts as the main downstream effector of TGFβ to modulate hypoxia’s effect on VSMC differentiation. Conclusion: Our results reveal a novel role of epicardial HIF in mediating coronary vasculogenesis by promoting their differentiation into VSMCs through noncanonical TGFβ signaling. These data elucidate that patterning of the coronary vasculature is influenced by epicardial hypoxic signals.

## 1. Introduction

Mesenchyme production from the epicardium is a critical developmental event during the generation of coronary vessels [1]. While the origin of coronary endothelial cells has been debated over the years, the consensus is that they arise from a heterogeneous lineage including the sinus venosus, epicardium, neural crest cells and the endocardium [2,3,4,5]. A heterogeneous lineage of the coronary vascular smooth muscle cells (VSMCs) is also expected, however it has been clearly shown that a subset of epicardium-derived cells (EPDCs) originate in the embryonic proepicardial serosa region, undergo epithelial-to-mesenchymal transition (EMT), invade the epicardial connective tissue and eventually the subcompact zone of the myocardium [6,7,8,9,10]. EPDCs are considered an important multipotent progenitor population that is able to generate these various cell lineages associated with coronary vessels [11,12]. In adults, the epicardial cells can also differentiate into various cell types including fibroblasts, endothelial, cardiomyocyte and VSMCs [13,14,15,16,17,18,19]. 

While many signaling pathways are involved in the development of the coronary vasculature, we and others have shown transforming growth factor-beta (TGFβ) is required for the proper development of coronary vessels by controlling EMT and further differentiation of epicardial cells into VSMCs [20,21,22,23,24,25,26,27,28]. TGFβ1–3 are a set of pleiotropic cytokines involved in the proliferation, differentiation, migration and EMT of various cell types and the signaling is transduced to the nucleus through the formation of heterodimeric receptor complexes between Type I [TβRI, also termed activin receptor-like kinase-5 (ALK-5)] and Type II (TβRII) II serine/threonine kinase receptors [29]. ALK5 phosphorylates and thereby activates specific SMADs (homologues of *Caenorhabditis elegans* protein Sma and the Mad (mothers against decapentaplegic) Drosophila protein), which then form heteromeric nuclear complexes with SMAD4. The resulting complexes regulate target genes ultimately serving as canonical transcriptional regulators of TGFβ. The response of this pathway to environmental signals likely contributes to the spatiotemporal specificity of coronary differentiation.

Tissue hypoxia has been shown in a range of contexts to drive adaptive gene expression. Many cellular adaptations to hypoxia during normal mammal embryogenesis are mediated by Hypoxia Inducible Factors (HIFs) which promote the transcription of multiple genes including those that support angiogenesis, energy metabolism and red blood cell production [30,31]. HIFs are heterodimeric basic-helix-loop-helix-Per-Arnt-Sim (bHLH-PAS) transcription factors containing oxygen-sensitive α-subunits that are readily degraded in the presence of oxygen (O_2_). Once stabilized under hypoxia, HIF-α subunits translocate to the nucleus and dimerize with its β-subunit which then activate multiple global and tissue- specific gene targets [31]. Our laboratories reported that particular cardiac regions (e.g., atrioventricular junction [AVJ] and interventricular septum [IVS]) where major coronary vessels emerge, express nuclear-localized HIF-1α and are highly hypoxic [32,33,34]. Alteration of HIF-1α expression by changing environmental oxygen levels or inducing expression of constitutively active HIF-1α (caHIF-1α) impaired coronary vessel patterning and created a variety of coronary anomalies some of which resemble those observed in patients [35,36]. Increased or decreased expression of Cited2, a regulator of HIF-1α caused abnormal coronary vasculature patterning and permeability [37,38]. We have also shown that forced expression of caHIF-1α in the epicardium of avian embryos resulted in reduced invasion of these cells into the myocardium due to the increased expression of the antagonistic receptor to Vascular Endothelial Growth Factor (VEGF), VEGFR1, which inhibits VEGF signaling [39]. These findings support multiple roles for HIF-1α in coronary development, homeostasis and diseases requiring further investigation.

Because VSMCs are critical for the growth, remodeling and homeostasis of vessels and also involved in cardiovascular pathogenesis (reviewed in [15]), it is important to reveal the functional mediators of epicardial mobilization and the steps involved in their differentiation in the context of the developing epicardium. It was recently reported that hypoxia induces the developmental differentiation of Tbx18+ epicardial cells to VSMCs through Snail [40]. As molecular hypoxic signals are key regulators in differentiation of various stem cell types, we postulated that hypoxia influences the development of the coronary vasculature in part by controlling the plasticity of epicardial cells. In the present study, we sought to identify alternate mechanisms by which hypoxia promotes their differentiation focusing on TGFβ that is strongly implicated in epicardial EMT and differentiation. We previously published that TGFβ-1 or TGFβ-2 induces EMT and smooth muscle differentiation in epicardial cells [21]. In addition, activation of TGFβRIII accesses the Par6/Smurf1/RhoA pathway to mediate epicardial cell invasion [28,41]. Inhibition of p160 rho-kinase (p160RhoK) and rhoA blocks EMT and prevents the appearance of calponin and SMA-positive cells. Our initial studies showed that the specific differentiation of epicardial cells with TGFβ1 into VSMCs was not disrupted by the forced expression of caHIF-1α [39]. Herein, we demonstrate that hypoxia influences RhoA/ROCK through TGFβ signaling to stimulate the differentiation of EDPCs into vascular smooth muscle cells. These observations provide a novel link between microenvironmental and growth factor mediated signals to regulate coronary vascular differentiation. Revealing the mechanisms controlling coronary development could direct the design of new diagnostic therapies and treatments related to cardiovascular anomalies and diseases.

## 2. Materials and Methods

### 2.1. Cell Culture

Immortalized epicardial cell lines, isolated from 13.5 dpc *Sm22α-lacZ* mice, crossed with the *ImmortoMouse* line were maintained at 33 °C in DMEM containing 10% FBS (Atlanta Biologicals, Flowery Branch, GA, USA), insulin-transferrin-selenium (ITS; Invitrogen, Grand Island, NY, USA) and 10 units/mL mouse gamma interferon (Peprotech, Rocky Hill, NJ, USA). Experimental cells were transferred to 5% FBS DMEM medium and cultured at 37 °C as previously described [21]. Hypoxia was achieved by culturing cells in 1% oxygen conditions.

### 2.2. Growth Factors and Inhibitors

TGFβ1 (Peprotech) was reconstituted in 1 mM citric acid/0.1% BSA and used at 250 pM. SB431452 (Sigma-Aldrich, St. Louis, MO, USA), SB202190 (Sigma-Aldrich), LY294002 (Calbiochem San Diego, CA, USA), UO126 (Sigma-Aldrich), Y27632 (Sigma-Aldrich) were used at 2.5 μM, 10 μM, 30 μM, 10 μM and 10 μM, respectively.

### 2.3. Transfection and Virus Infection

Contends Cells were cultured on collagen I coated 35 mm dishes at 37 °C and transfected with 0.1 nmol siRNA (Invitrogen) and 8 Μl Xtreme siRNA transfection reagent (Roche, Pleasanton, CA, USA). Medium was changed after 24 h before proceeding with other treatments.

Prior to adenovirus infection, cells were plated with Opti-MEM I (Gibco, Thermo Fisher Scientific, Gaithersburg, MD, USA) reduced serum medium containing adenovirus (1 × 10^9^ PFU/mL GFP control (Ad-GFP) or dominant negative (Ad-dnRhoA) co-expressing GFP) overnight at 37 °C. Cells were then transferred to 5% FBS DMEM medium and further cultured under hypoxic conditions before immunohistochemical analysis. Similarly, cells were incubated with serum-reduced medium containing polybrene and lentiviral particles overnight and changed to 5% FBS DMEM before further analysis.

### 2.4. Measurement TGFβ1, RhoA and lacZ Activity

Epicardial TGFβ1 (R&D Systems, Minneapolis, MN, USA) and RhoA (Cytoskeleton Inc., Denver, CO, USA) levels were quantified following the manufacturers’ instructions. *Sm22α-lacZ:Immorto* epicardial cells were assayed as previously described with the Galacto-Light Plus system (Applied Biosystems, Foster City, CA, USA) [21].

### 2.5. Immunohistochemistry

Epicardial cells were plated on collagen-I coated chamber slides (BD Biosciences). Before the staining, cells were washed in PBS and fixed with 4% PFA for 10 min. at room temperature. Cells were further permeabilized with 0.2% TritonX-100 for 5 min and blocked with 2% BSA in PBS for 1 h. After blocking, cells were incubated with primary antibodies overnight at 4 °C and detected with appropriate secondary antibodies. Photomicrographs were captured with an inverted fluorescence microscope (Leica DM2500, Leica Microsystem, Buffalo Grove, IL, USA) and QCapture Pro software (Version 5.1, QImaging, Surrey, British Columbia, Canada). The following antibodies were used: anti-ZO1 (1:100, Invitrogen), anti α-smooth muscle actin (1:200, Sigma-Aldrich), anti-Smad2 (1:100, Cell Signaling, Danvers, MA, USA).

### 2.6. Western Blotting

Equal amounts of protein samples quantified by a Bradford assay (Pierce, Thermo Fischer Scientific, Gaithersburg, MD, USA) were loaded onto 10% SDS-PAGE gels (Bio-Rad Laboratories, Hercules, CA, USA) and transferred to a PVDF membrane (Millipore Sigma, Burlington, MA, USA). Membranes were blocked for 1 h at room temperature with 5% BSA in followed by incubation with primary antibodies overnight at 4 °C. Primary antibodies against the following proteins were utilized: SMAD2 (1:1000, Cell Signaling), SMAD4 (1:1000, Cell Signaling) phospho-SMAD2 (1:1000, Cell Signaling) phosphor-AKT (1:2000, Cell Signaling), AKT (1:1000, Cell Signaling), phospho-42/44 (1;1000, Cell Signaling), 42/44 MAP kinase (1:1000, Cell Signaling), β actin (1:100,000, Sigma-Aldrich), α-smooth muscle actin (1:2000, Sigma-Aldrich), HIF-1α (1:600, gift of Dr. Faton Agani from Hypoxis Bioscience), SM22α (1:4000, Abcam, Cambridge, UK) and phospho-TGFβRII (1:200, Santa Cruz Biotechnology Inc., Dallas, TX, USA). After washing, blots were incubated with a secondary horseradish peroxidase-linked antibodies (Cell Signaling) for 1 h at room temperature at a dilution of 1:5000. Signals were visualized with a HRP detection kit (Pierce, Thermo Fischer Scientific, Gaithersburg, MD, USA).

### 2.7. Real Time RT-PCR

cDNA was synthesized from total RNA isolated from cultured epicardial cells. Genes were amplified with Universal SYBR Green Master (Roche) by Real-time PCR (Applied Byosystems StepOne Plus, Thermo Fisher) and quantified by the ^∆∆^C_T_ method using 18S as the internal control, as previously described [39]. Primer sequences are listed in Table 1.

### 2.8. Statistical Analyses

All experiments were executed in triplicate. Data are represented as mean ± S.E.M. Statistical analyses were performed using student’s paired *t* test. *p* value < 0.05 was considered significant.

## 3. Results

**Figure 1:**
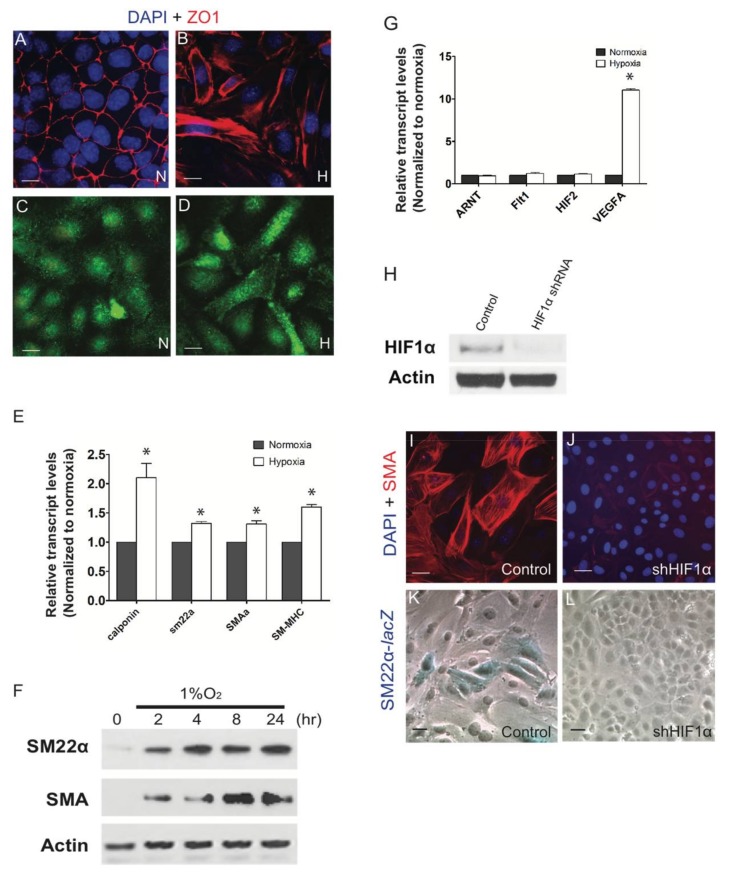
Hypoxia promoted vascular smooth muscle cell (VSMC) differentiation in epicardial cells. Immortalized mouse epicardial cells (EPDCs) were cultured under normoxic (21% O_2_) or hypoxic (1% O_2_) conditions for 24 h. In response to hypoxia, EPDCs underwent epithelial-mesenchymal transformation (EMT). Loss of cell to cell contact was evident in the zonula occludens-1 (ZO-1) labeled cells (**A**,**B**) but with no significant alterations in the expression of periostin following hypoxic treatment (**C,D**). Real-Time PCR (*n* = 3) (**E**) and Western blot (**F**) showed the induction of vascular smooth muscle cell markers, α-smooth muscle actin (SMA) and smooth muscle protein 22-alpha (SM22α) under hypoxic conditions. Hypoxia did not alter the expression of hypoxia-related genes *Arnt* or *Hif-2α* nor *Flt-1* but induced the expression of a HIF transcriptional target, *VefgA* (*n* = 3) (**G**) HIF-1α decreased in lysates from hypoxic EPDC cultures infected with small hairpin RNA (shRNA) against HIF-1α (shHIF-1α). Scramble shRNA was used as a negative control (**H**). shHIF-1α repressed expression of SMA and Sm22-lacZ activity (**I**–**L**) following 24 h at 1% O_2_. * *p* < 0.05; Bar: 50 μm.

Differentiation of many cell types, including epicardial cells, requires epithelial–mesenchymal transformation (EMT). Since we previously reported that the forced expression of activated HIF-1α ex vivo or in ovo in the epicardium of developing avian embryos promoted epicardial EMT, we used a well-established immortalized mouse EPDC culture system to closely examine the contribution of hypoxia during these developmental events. We first examined the effects of hypoxia on EMT, expecting the loss of epithelial characteristics and the acquisition of mesenchymal cell properties including the loss or redistribution of proteins associated with cell-cell junctions. In comparison to control EPDC cultures that maintain a compact, cobblestone epithelial-like appearance, cultures exposed to hypoxic conditions at 1% oxygen (O_2_) exhibited an elongated cellular morphology at 24 h. (Figure 1A–D). We examined the distribution of zona occludens protein 1 (ZO-1), which interacts with the actin cytoskeleton and marks epithelial tight junctions. Epicardial normoxic cultures showed strong cell surface localization of ZO-1 in regions of cell-cell contact. In contrast, hypoxic treatment resulted in the redistribution of ZO-1 indicating detachment of cells from each other, a feature for EMT [42] (Figure 1A,B).

During development, EMT can be further accompanied by the differentiation of epicardial cells into various cell types. By qRT-PCR we observed that hypoxia induces the expression of transcripts associated with VSMC including Calponin, Smooth Muscle Protein 22-α (SM22α), α-Smooth Muscle Cell Actin (SMA) and SM myosin heavy chain (SM-MCH) (Figure 1E). Consistent with transcriptional changes, SM22α and SMA proteins in EPDCs were detected following 2 h of 1% O_2_ treatment (Figure 1F). In contrast, we observed no hypoxic effect on the expression of periostin, shown to be highly expressed in EPDCs and in cardiac fibroblasts (Figure 1C,D) [42].

Observing that hypoxia induces EMT and differentiation of EPDCs, we sought to determine the direct effects of HIF signaling on this process. We used our SM22α-lacZ mouse reporter EPDC line that serves as a model for differentiation [21]. To abrogate HIF’s transcriptional activity, we knocked down HIF-1α by infection of lentivirus expressing short hairpin RNA for HIF-1α (shRNA, gift from Dr. Yu-Chung Yang) or control-scrambled shRNA (Figure 1H). At 24 h of 1% O_2_ treatment we did not observe any cell death in either cultures. However, silencing HIF-1α reduced the expression of smooth muscle cell markers in EPDC cultures as SM22-lacZ reporter (Figure 1I–L) or of α-SMA expression was reduced (Figure 1K,L). These results imply that hypoxia promotes the differentiation of epicardial cells through the transcriptional activity of HIF-1α.

**Figure 2:**
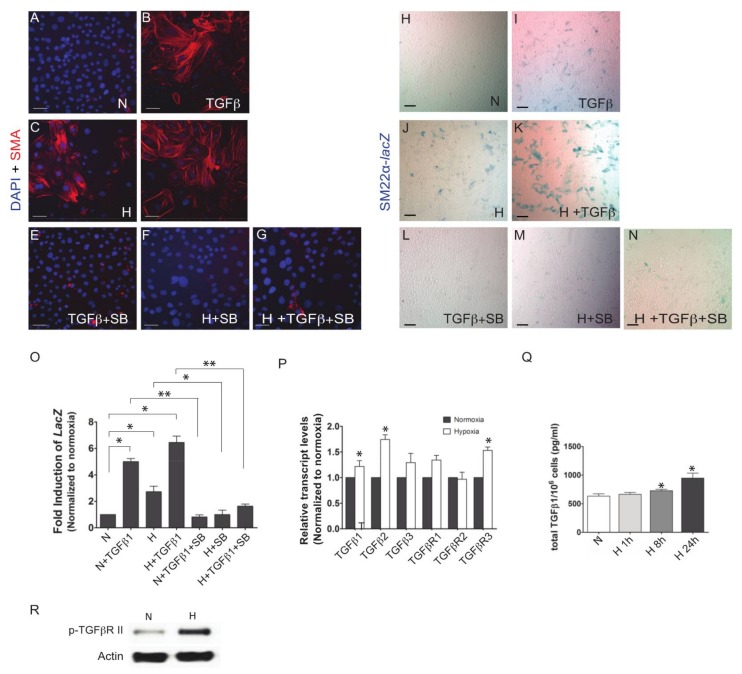
The TGFβ pathway is involved in the hypoxic induction of VSMC differentiation in EPDC cultures. ALK5/TGFβR I inhibitor SB431542 (SB) abolished the hypoxia-stimulated (1% O_2_, 36 h) expression of SMA (**A**–**G**) and SM22α-lacZ (**H**–**N**) in EPDC cultures similar to its effect on cultures treated with 250 pM TGFβ1. SM22-lacZ activity was assayed to quantify the extent of cell differentiation. Data are presented as mean fold induction of triplicate samples ± SEM compared to normoxia only conditions (*n* = 3) (**O**). Hypoxia induced *TGFβ-1* and *-2* and *TGFβ*-*R3* transcript levels (*n* = 3) (**P**). ELISA data indicated that expression of TGFβ1 ligand in epicardial cells increased significantly under hypoxia by 8 and 24 h (*n* = 3) (**Q**). Hypoxia up-regulated the phosphorylation of TGFβRII (1% O_2_, 8h) (**R**). N: Normoxia; H: Hypoxia; SB: SB431542. * *p* < 0.05, ** *p* < 0.001; Bar: 50 μm.

We had previously shown that TGFβ-induced EMT and VSMC differentiation in EPDCs required the downstream kinase activity of ALK5 [21]. Treatment of EPDCs with 250 pM TGFβ1, hypoxic treatment, or both resulted in increased numbers of SMA^+^ and SM22-LacZ^+^ cells (Figure 1A–D and Figure 2H–K). Selective inhibition of transforming growth factor-beta (TGF-β) superfamily type I activin receptor-like kinase (ALK) receptors [SB: 2.5 μM SB431542] blocked the differentiation of EPDCs in all conditions (Figure 2E–G,L–N). To quantify the differentiation of EPDCs, we measured LacZ expression in Sm22α-lacZ immortalized epicardial cells. Relative to control normoxic EPDCs, LacZ expression is 5- and 3-fold greater in TGFβ1 or hypoxic treated cultures, respectively. Addition of ALK5 kinase inhibitor (SB431542) impaired the ability of TGFβ1 or hypoxic treated epicardial cells to induce Sm22-LacZ expression (Figure 2O). These findings support that the ability of hypoxia to promote VSMC differentiation is mediated via the TGFβ pathway.

Since hypoxia is known to induce the secretion of cytokines and growth factors in various cell types we next sought to determine its effects on key components of the TGFβ pathway. Transcript levels for TGFβ-1 and -2 and TGFβR3 are increased in epicardial cultures following 24 h of hypoxic treatment (Figure 2P). Conditioned media collected from EPDCs cultured for 8 and 24 h at 1% O_2_ had increased levels of total TGFβ-1 as measured by ELISA (Figure 2Q). Furthermore, hypoxia induced the phosphorylation of TβRII, previously shown to be required and sufficient to drive EMT and VSMC differentiation in epicardial cells (Figure 2R).

**Figure 3:**
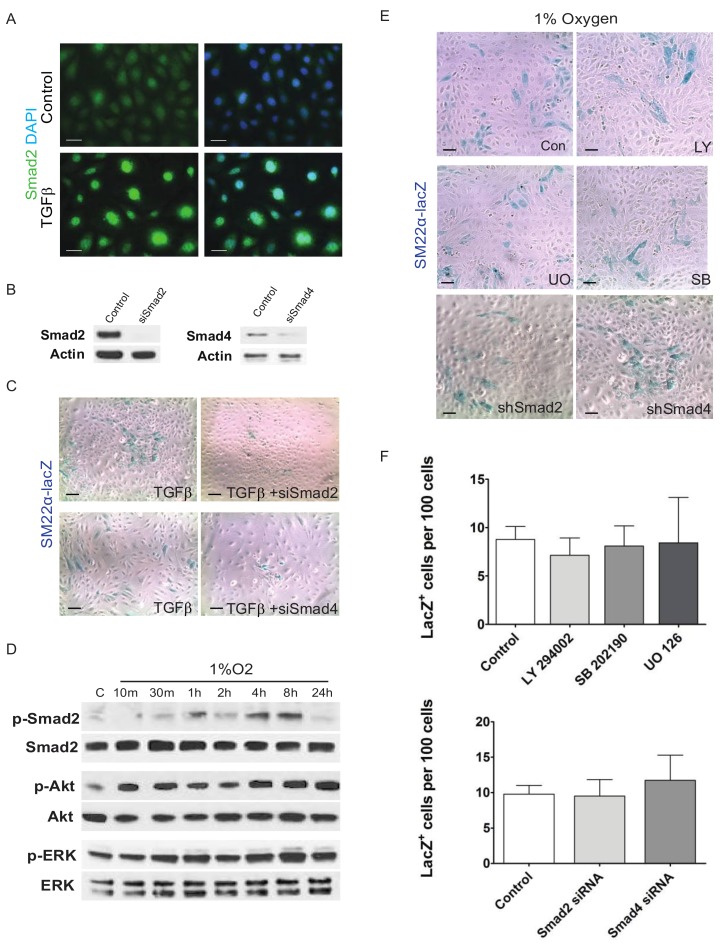
Hypoxia mediated activation of SMADs is dispensable for differentiation of epicardial cells into VSMCs. EPDCs cultures treated with 250 pM TGFβ-1 induced nuclear-localization of Smad2, indicative of its activation (**A**). Western blots of EPDCs supported the knockdown of SMAD2 or SMAD4 using the appropriate siRNAs (**B**), which reduced the generation of SM22α-LacZ cells in TGFβ-1 treated EPDC cultures (**C**). Hypoxia triggered the phosphorylation of Smad2, Akt and ERK (**D**). Inhibition of PI3K/AKT (LY: 30 μM LY294002), MEK1/2 (UO: 10 μM UO126), or p38 MAPK (SB: 10 μM SB202190) pathways did not block hypoxia induced smooth muscle cell differentiation of EPDC cultures. Similarly, knockdown siSMAD2 or siSMAD4 also failed to block smooth muscle cell differentiation in hypoxia treated EPDC cultures (**E**). Quantification of SM22-LacZ^+^ in hypoxic EPDC cultures treated with chemical inhibitors or siRNAs showed no significant differences (*n* = 3) (**F**). Bar: 50 μm.

TGFβ signaling is also known to mount multiple responses. In fact, non-SMAD mediated pathways are associated with TGFβ’s ability to induce EMT [43,44,45]. By Western blotting, we observed that hypoxia induced and sustained pERK and pAKT activation for 24 h in EPDCs (Figure 3A). However, treatment with specific inhibitors for PI3K/AKT (LY294002), p38 MAPK (SB202190), or MEK1/2 (UO126) pathways did not block the hypoxia induced expression of Sm22-LacZ in these epicardial cultures (Figure 3E,F).

We next sought to examine the effects of hypoxia downstream of TGFβ receptor activation by examining the activation of SMAD transcription factors. As transcription factors, SMADs have been shown to promote the differentiation of neural crest cells into VSMCs [46]. Canonical signaling by Alk5 involves SMAD2 and SMAD3 and results in their phosphorylation and nuclear translocation. As a control, we observe that TGFβ-1 stimulates the nuclear translocation of SMAD2 (Figure 3B) and knockdown of either SMAD2 or SMAD4 inhibits the differentiation of epicardial cells (Figure 3A). Thirty min after 1% O_2_ treatment, phospho-SMAD2 levels varied over a 24 h period (Figure 3B). To examine if SMAD signaling is required for epicardial transformation into smooth muscle cells, we transfected immortalized Sm22-lacZ epithelial cells with interfering small double-stranded RNA to knock down either SMAD2 or SMAD4 (Figure 3C). We found that knocking down SMAD signaling inhibited the ability of TGFβ to induce VSMC differentiation (Figure 3D) but did not affect the ability of hypoxia to do the same (Figure 3E).

**Figure 4:**
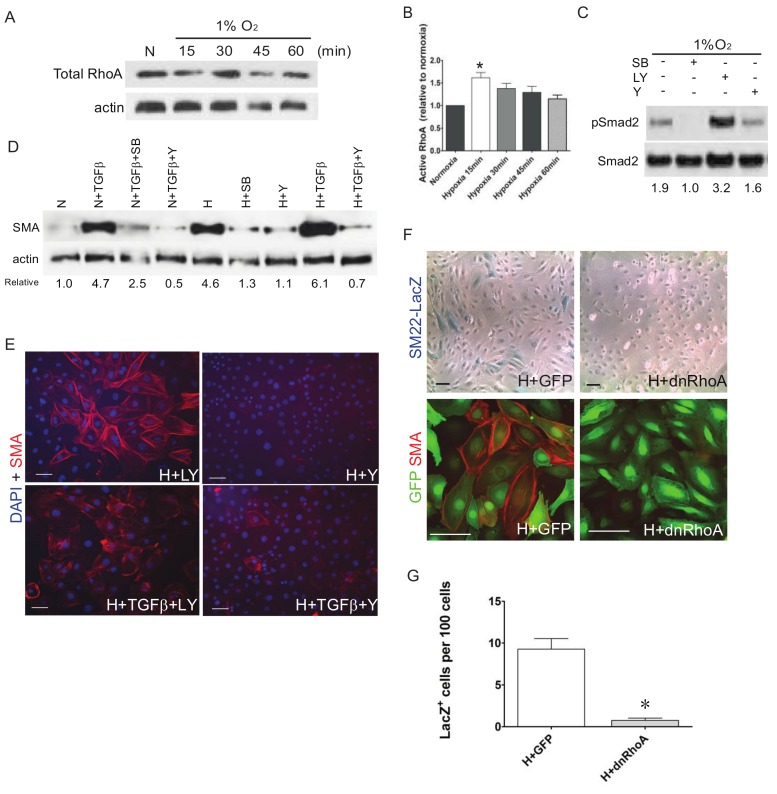
The RhoA/ROCK pathway acts as the main downstream effector of TGFβ to regulate VSMC differentiation in hypoxic EPDC cultures. Western blot results indicate that total RhoA levels in EPDCs are not significantly altered by hypoxia (**A**) but the RhoA activation (RhoA-GTP), measured by ELISA, was induced in as early as 15 min (**B**). phospho-SMAD2 activation by hypoxia in EPDC cultures was inhibited by selectively blocking ALK5/TGFβR I (SB; SB421542) but not with inhibitor to PI3K/AKT (LY: 30 μM LY294002) and found to increase with inhibition to ROCK (Y: 10 μM Y27632) (*n* = 3) * *p* < 0.05) (**C**). In contrast, inhibition of ROCK (Y) blocked SMA expression stimulated by either TGFβ (250 pM) or hypoxia, similar to blocking ALK5/TGFβR I receptor (SB: SB431542) (**D**). Expression of SMA in hypoxic EPDC cultures was significantly compromised by inhibiting ROCK (**Y**) but not PI3K/AKT (LY), even in cultures treated with exogenous TGFβ (**E**). Compared with Ad-GFP treated control cultures, SM22 and SMA smooth muscle cell markers were abolished in EPDCs infected with adenovirus expressing dominant negative RhoA (Ad-dnRhoA) (**F**). Quantification shows significantly decreased LacZ^+^ cell numbers in Ad-dnRhoA than Ad-GFP control treated hypoxic EPDCs cultures (*n* = 3) * *p* < 0.05 (**G**). N: Normoxia; H: Hypoxia; Bar: 50 μm.

TGFβ can also induce activation of Rho-like GTPases that, among their various roles, control the dynamic cytoskeletal organization of cells including actin remodeling during EMT. It has been previously shown that RhoA is required for VSMC differentiation from proepicardial cells downstream of TGFβ signaling [47,48]. While total RhoA levels are not altered by 1% O_2_ treatment in EPDC cultures (Figure 4A), it significantly induces the activation of RhoA measured by ELISA (Figure 4B). Western blot data indicate that unlike ALK5/TGFβR I inhibition (SB), specific blockade of RhoA signaling in EPDCs with ROCK inhibitor (Y) does not reduce the hypoxic induction of phospho-SMAD2 (Figure 4C). However, inhibition of ROCK (Y) reduces the enhanced expression of SMA by either TGFβ and/or hypoxic treatments, similar to the effects of blocking ALK5/TGFβR I (SB), in contrast to inhibiting AKT/PI3K (LY) (Figure 4D,E). To confirm the involvement of ROCK, EPDC cultures were infected with a dominant-negative RhoA (Ad-dnRhoA) which hampered hypoxia induced expression of SM22-LacZ or SMA compared to control untreated or green fluorescent protein (Ad-GFP) infected EPDCs (Figure 4F,G). There results argue that hypoxia influences EMT and differentiation of EPDCs, partially through TGFβ-mediated noncanonical signaling through RhoA/ROCK.

## 4. Discussion/Conclusions

**Figure 5:**
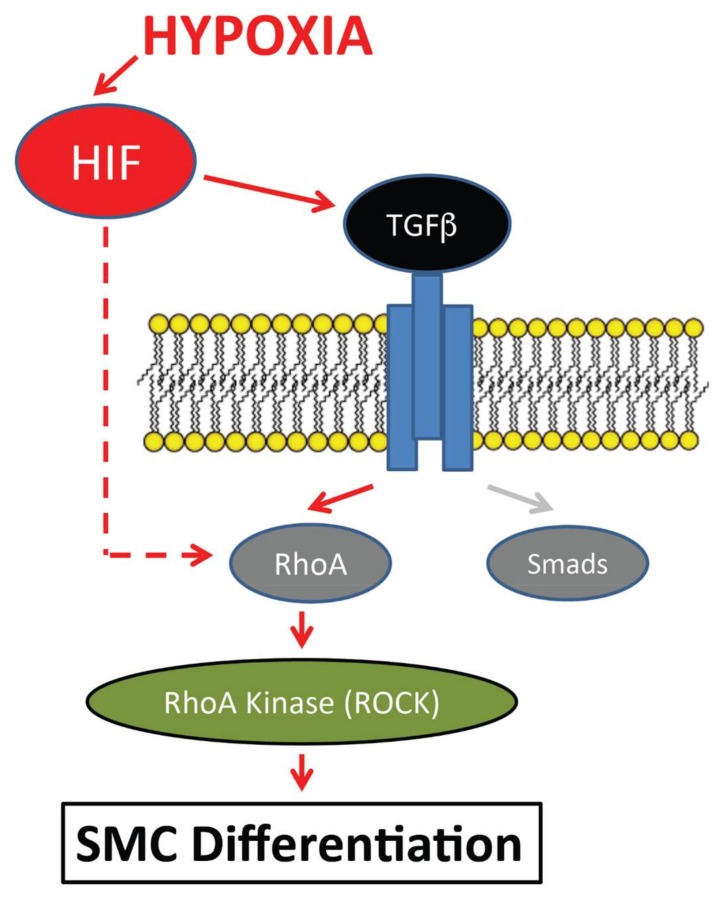
Hypoxia positively regulates epicardial cell differentiation into VSMC by the activation of the TGFβ pathway. Hypoxia promotes RhoA as the major downstream regulator of the TGFβ pathway affecting EPDC differentiation into VSMCs.

During early steps in the development of the coronary vasculature, endothelial tubes connect and form a capillary plexus or anastomosis that eventually transitions into the extensive branching network of the mature coronary vessels. This extensive remodeling requires the envelopment of the coronary endothelial cells by smooth muscle cells. Here, we demonstrate that hypoxia plays a critical role in regulating key steps in the differentiation of epicardial cells into vascular smooth muscle cells. Our findings showed that low oxygen conditions enhanced EMT characteristics of epicardial cell cultures and promoted their differentiation into VSMCs. Our results also revealed an important and novel functional connection between hypoxia and TGFβ-mediated EMT and differentiation of EPDCs into VSMCs through the activation of the RhoA/ROCK pathway independent of SMADs (Figure 5).

The development of coronary vessels during embryogenesis requires regulated epithelial-mesenchymal transition, cell migration and differentiation of a subset of epicardial cells. We show that epicardial EMT stimulated by low oxygen tension requires HIF-1α, a primary transcriptional mediator of responses to hypoxia, supporting our previous finding showing that constitutive HIF-1α (AdcaHIF1α) mediated EPDC differentiation and induced EMT in primary embryonic quail epicardial cells while hypoxic treatment promoted the expression of ZO-1 in an immortalized EPDC line [36,39]. We also demonstrated that caHIF1α infected primary EPDCs were capable of differentiating into SMA^+^ cells in response to TGFβ-1 treatment [36,39]. The epicardium has been identified as a hypoxic microenvironment expressing Hif-1α and harboring progenitor cells during embryogenesis and in adults [39,49,50,51]. Supporting the present observations, Jing et al. [40] recently showed that hypoxia induced the differentiation of TBX18^+^ epicardial cells to undergo EMT and differentiate into VSMCs through HIF-mediated activation of Snail (a major regulator of EMT) that is also known to be induced by TGFβ [52,53,54]. However, in vivo loss of epicardial Snail1 did not lead to any aberrant morphogenetic processes [55]. In the avian epicardium specifically, we showed that Snail over-expression induced epicardial EMT in vitro and enhanced EPDC invasion into the myocardium in vivo [56]. We also demonstrated that HIF-1α promotes the expression of another EMT-related transcription factor, Twist2, associated with the downregulation of E-cadherin that is important to allow epicardial EMT [39]. Thus, hypoxia and TGFβ may control the mobilization of various EMT inducing transcription factors.

As it has been shown that TGFβ signals play a central role in inducing mesenchymal transformation of the epicardium, we hypothesized that the influence of hypoxia on EPDC differentiation occurs via this molecular pathway [20,25,57,58]. While hypoxic treatment induced the expression of TGFβ-1, TGFβ-2 and TGFβ-RIII along with the activation of TGFβ-RII in EPDC cultures, the inhibition of ALK5/TGFβR I suppressed hypoxia-mediated SM22 and SMA induction, consistent with this pathway being required for EPDC differentiation and indicating that this is the primary pathway responsible for EPDC differentiation [23,24].

There are multiple mechanisms by which hypoxia could modulate the specific TGFβ downstream signals to induce differentiation of EPDCs. Hypoxia has been shown to stimulate epithelial to mesenchymal transition (EMT) in other cell types including renal tubular cells [59] and hepatocytes [60] in part by mediating the TGFβ pathway. In response to hypoxia, we observed the phosphorylation of SMAD2 as early as 30 min, comparable to the response to TGFβ treatment of neural crest cells that also differentiate into VSMC [61]. However, inhibition of the canonical TGFβ pathway by knocking down of SMAD-2 or -4 had no effect on the ability of hypoxia to induce differentiation of EPDCs. While loss of TGFβ-RIII accompanies coronary vessel anomalies, we recently identified dysregulation of NF-kB signaling in TGFβ-RIII knockout epicardial cells [23,24]. Interestingly, important crosstalk between HIF and NF-kB signaling previously demonstrated in immune cells may relate to the increased *TGFR3* transcript levels we observed in our hypoxic epicardial cell cultures [62].

EMT induction by hypoxia is consistent with previous data indicating that TGFβ-induced EMT and VSMC differentiation in EPDCs was dependent on RhoA [21,48]. In EPDC cultures, hypoxia did not change the total expression of RhoA but did induce RhoA activation in EPDCs, similar to what has been observed with TGFβ treatment alone [48,61]. Interestingly, while inhibition of ALK5/TGFβR I blocked the activation of SMAD2 by hypoxia, SMAD2 activation was not affected by the inhibition of ROCK/RhoA under the same conditions. In fact, we observed that inhibition of PI3K/AKT further increased the hypoxic induction of SMAD2. Thus, hypoxia may play singular roles in mediating alternative TGFβ pathways in a cell specific manner. In epicardial cells it appears that it controls the activation of RhoA which is associated with their transformation into coronary VSMCs. It has been demonstrated that hypoxia induces RhoA and its activation and that *RhoA* and *Rock1* genes are direct HIF targets [63,64,65]. Using chemical and genetic approaches to block the downstream RhoA target Rho kinase (ROCK) led to the decreased expression of either hypoxia-induced SMA or SM22 expression in EPDCs, much the same way that inhibition of RhoA blocked the differentiation of neural crests into SMCs [61]. In all, it appears that hypoxic signals tightly control various components of the TGFβ pathway to regulate this developmental process through RhoA/ROCK (Figure 5). In the present model system, our ex vivo EPDC differentiation system had a significant influence in the promotion of VSCMs. Hypoxia is a specific micro-environmental cue that has a variety of effects on the maintenance and differentiation of cells. However, the multifaceted cardiac milieu influences the controlled differentiation of EPDCs into various other cell types, including cardiac fibroblasts, to support the coronary vasculature crucial for proper cardiac function. Indeed, multiple transcriptional pathways are required for functional epicardial development, which may most effectively work in trans- or cis- [52].

Importantly, HIF is also known to be upregulated in ischemic tissues as a result of reduced oxygen concentrations as in the case of myocardial infarction [66,67,68,69,70]. Emerging data suggests that under conditions reproducing the hypoxic embryonic environment cells in the adult heart may be reactivated to undergo EMT and migrate to incorporate into new coronary vessels to stimulate the revascularization and regeneration of the injured heart [17,71,72,73,74,75]. Although TGFβ has been shown to induce EMT and cell differentiation in human adult epicardial cells, differentiation was not mediated by RhoA/ROCK [76]. Together, these results support a model whereby hypoxia and TGFβ activate the RhoA/ROCK pathway to promote the differentiation of EPDCs into coronary VSMCs during development but also show the necessity of further studies to examine in more detail how hypoxia orchestrates the molecular signals associated with the behavior of adult epicardial cells that could help in the development of cardiovascular therapies in response to hypoxic signals following cardiac injury.

## Figures and Tables

**Table 1:** Primer Sequences.

Name	Sequence
TGFβ1 Forward	CCGCAACAACGCCATCTATG
TGFβ1 Reverse	CCCGAATGTCTGACGTATTGAAG
TGFβ2 Forward	AGAATCGTCCGCTTTGATGTC
TGFβ2 Reverse	TCTGGTTTTCACAACCTTGCT
TGFβ3 Forward	GGACTTCGGCCACATCAAGAA
TGFβ3 Reverse	TAGGGGACGTGGGTCATCAC
TGFβR1 Forward	ATATCTGCCATAACCGCACTG
TGFβR1 Reverse	AAAGGGCGATCTAGTGATGGA
TGFβR2 Forward	CCTCACGAGGCATGTCATCAG
TGFβR2 Reverse	ACAGGTCAAGTCGTTCTTCACTA
TGFβ3 Forward	GGACTTCGGCCACATCAAGAA
TGFβ3 Reverse	TAGGGGACGTGGGTCATCAC
TGFβR1 Forward	ATATCTGCCATAACCGCACTG
TGFβR1 Reverse	AAAGGGCGATCTAGTGATGGA
TGFβR2 Forward	CCTCACGAGGCATGTCATCAG
TGFβR2 Reverse	ACAGGTCAAGTCGTTCTTCACTA
TGFβR3 Forward	CATCTGAACCCCATTGCCTCC
TGFβR3 Reverse	CCTCCGAAACCAGGAAGAGTC
Sm-22α Forward	AGCCAGTGAAGGTGCCTGAGAAC
Sm-22α Reverse	TGCCCAAAGCCATTAGAGTCCTC
SMAα Forward	GAGAAGCCCAGCCAGTCG
SMAα Reverse	CTCTTGCTCTGGGCTTCA
Calponin Forward	GAAGGCAGGAACATCATTGGACTG
Calponin Reverse	CTCAAAGATCTGCCGCTTGGTGCC
SM-MHC Forward	GGCTTCATTTGTTCCTTCCA
SM-MHC Reserve	CGAGCGTCCATTTCTTCTTC
